# Outcomes of Early Versus Delayed Definitive Surgical Intervention in Multisystem Trauma Patients: A Systematic Review of Timing Strategies in General Surgery Emergencies

**DOI:** 10.7759/cureus.90136

**Published:** 2025-08-15

**Authors:** Mazin Osman, Khald Alhamoud Almatar, Fahad Almatar, Shahd A Awad, Hind K Mohamed, Mohamed K Abouelsadat, Othman T Alamodi, Gorashi Humida Ali Gorashi, Albina Mercy, Mawada Taha, Manahil Awan

**Affiliations:** 1 General Surgery, British United Provident Association, Jeddah, SAU; 2 Surgery, Royal Liverpool University Hospital, Liverpool, GBR; 3 General Practice, Victoria Medical Group, North York, CAN; 4 General Surgery, Dallah Hospital, Riyadh, SAU; 5 General Surgery, Farwaniya Hospital, Sabah Al Nasser, KWT; 6 Pediatric Surgery, University of Benghazi, Benghazi, LBY; 7 Vascular Surgery, Royal Free Hospital, London, GBR; 8 General Practice, Fakeeh Care Group, Jeddah, SAU; 9 Anatomy, University of Khartoum, Khartoum, SDN; 10 Internal Medicine, Davao Medical School Foundation Inc., Davao City, PHL; 11 General Surgery, The National Ribat University, Khartoum, SDN; 12 Surgery, Liaquat National Hospital, Karachi, PAK

**Keywords:** delayed surgery, early surgical intervention, general surgery emergencies, multisystem trauma, trauma outcomes

## Abstract

This review explores how the timing of surgery affects outcomes in adults with multisystem trauma requiring general surgical or orthopedic procedures. A targeted search of PubMed and other major databases up to July 2025 identified six relevant studies, including four observational cohorts, one meta-analysis, and one narrative review, collectively covering 273,683 patients. Early definitive surgery, performed within 24-48 hours of admission, was associated with lower mortality rates, fewer respiratory complications, and shorter ICU and hospital stays compared to delayed interventions. One large study reported no significant mortality difference, highlighting the importance of preoperative stabilization and individualized care. Despite variability in study design and the definition of “early” surgery, current evidence suggests that early operative management in stable trauma patients, including abdominal trauma, offers meaningful benefits in reducing complications and improving recovery.

## Introduction and background

Trauma is a major global health concern, claiming close to five million lives annually and accounting for nearly 10% of all global deaths, with a disproportionate impact on young, economically active individuals [1]. Among trauma cases, multisystem injuries involving two or more critical organ systems, such as the abdomen, chest, or extremities, pose complications and challenges and require urgent, well-coordinated surgical interventions, including procedures such as laparotomy or splenectomy [2]. The timing of surgical intervention plays a pivotal role in determining patient outcomes. While advances in diagnostic imaging, resuscitation, and triage have improved early management, there are still no worldwide standards on the optimal timing for definitive surgery. Evidence suggests that operations performed within 24-48 hours of admission can limit systemic inflammatory responses, reduce infection risk, and improve overall physiological recovery by avoiding the cumulative burden of delayed intervention, often described as the “second-hit” phenomenon [3].

However, not all patients are candidates for early surgery. Those with unstable hemodynamics, uncontrolled bleeding, coagulopathy, or severe metabolic disturbances may require damage control strategies and staged resuscitation prior to undergoing definitive surgical repair [4]. This need for case-by-case decision-making complicates the development of standardized treatment protocols, especially in abdominal surgery. The situation is further compounded in low- and middle-income countries (LMICs), where almost 90% of trauma-related deaths occur. In these regions, delays are frequently linked to systemic barriers such as insufficient infrastructure, limited availability of trained personnel, and resource shortages, rather than the patient’s physiological condition [5]. These inequities highlight the challenges of applying early surgical strategies across varied healthcare systems.

Although many studies have examined the relationship between surgical timing and outcomes, the evidence remains fragmented and at times contradictory. There is a clear need for a comprehensive synthesis to evaluate how early versus delayed definitive surgical interventions influence mortality, postoperative complications, ICU and hospital stays, and overall recovery. This systematic review seeks to address this gap by comparing early and delayed surgical approaches in adults with multisystem trauma, aiming to provide actionable insights for clinical protocols and resource allocation.

## Review

Materials and methods

Review Design and Objectives

This systematic review was designed and reported in line with the Preferred Reporting Items for Systematic Reviews and Meta-Analyses (PRISMA) framework 2020 guideline [6]. The primary aim was to assess and compare clinical outcomes associated with early versus delayed definitive surgical interventions in adult patients with multisystem trauma requiring general surgical procedures. Since the review utilized data solely from previously published studies, ethical approval was not applicable. The analysis focused on outcomes most frequently reported across the included studies, namely, mortality, postoperative complications, length of stay in the ICU, total hospital stay, and overall morbidity. For consistency, early surgical intervention was defined as procedures performed within 24-48 hours of hospital admission, whereas surgeries conducted beyond this period were classified as delayed interventions. By integrating findings from four observational cohort studies, one meta-analysis, and one narrative review, this review aims to provide a clearer understanding of how surgical timing influences outcomes in multisystem trauma care.

Eligibility Criteria 

The inclusion criteria were structured according to the PICO (Population, Intervention, Comparison, Outcome) framework [7]. Eligible studies involved adult patients aged 18 years or older who sustained multisystem trauma and underwent general surgical procedures, such as laparotomy, bowel repair, or splenectomy. Early surgical intervention was defined as surgery performed within 24-48 hours of hospital admission, while delayed intervention referred to procedures conducted after 48 hours. Studies were required to report at least one key clinical outcome, including mortality, overall morbidity, ICU stay, total hospital length of stay, or postoperative complication rates. Only peer-reviewed studies conducted on human subjects and published in English between January 2000 and July 2025 were considered for inclusion. Studies were excluded if they focused on isolated injuries (e.g., head trauma or single-limb injuries), pediatric populations (patients under 18 years), or animal-based research. Additionally, non-peer-reviewed articles, case reports, small case series with fewer than 10 patients, narrative reviews, editorials, commentaries, and publications not in English were excluded from the review.

Information Sources and Search Strategy

A comprehensive literature search was conducted across PubMed, Google Scholar, Scopus, ScienceDirect, and Cochrane Library to identify relevant studies published up to July 2025. Additional manual searches of reference lists from included articles were performed to ensure completeness. The search strategy used combinations of Medical Subject Headings (MeSH) and keyword terms, such as “early surgery”, “delayed surgery”, “trauma”, “multisystem trauma”, “timing of surgery”, “emergency laparotomy”, “polytrauma”, and “general surgical emergencies”. Boolean operators (AND/OR) were used to refine the search. Filters were applied to restrict the results to English-language human studies. Preference was given to studies conducted in or applicable to LMICs to enhance relevance in resource-limited contexts.

Study Selection and Data Extraction

Two reviewers independently screened all titles, abstracts, and full-text articles, resolving any disagreements through discussion. Data extraction followed a structured template, capturing key information such as study design, sample size, demographics, timing of surgery, and outcomes. From 238 records initially identified, 58 duplicates were removed, leaving 180 studies for title and abstract screening. Of these, 140 were excluded due to irrelevant populations, lack of early versus delayed surgical comparison, or unrelated outcomes. Forty full-text articles were then assessed, with 34 excluded for reasons such as small sample size (<10 patients), non-general surgical procedures, reviews, or non-English publications. Ultimately, six studies fulfilled all eligibility criteria and were included in this review.

Risk of Bias Assessment

The risk of bias for the included studies was evaluated according to their respective designs. Randomized controlled trials (RCTs), where applicable, were assessed using the Cochrane Risk of Bias 2.0 tool [8], while non-randomized observational studies were appraised using the Risk of Bias in Non-randomized Studies of Interventions (ROBINS-I) tool [9]. The single narrative review was critically examined with the Scale for the Assessment of Narrative Review Articles (SANRA) criteria [10] to ensure methodological rigor and relevance. All evaluations were conducted independently by two reviewers, and any discrepancies were resolved by discussion and consensus. Overall, most studies demonstrated a low to moderate risk of bias, with common concerns related to variations in surgical timing definitions, trauma severity classification, and incomplete outcome reporting. However, all included studies were deemed to provide reliable and valuable evidence for the review.

Data Synthesis

Due to clinical and methodological heterogeneity across the included studies, including variations in patient demographics, injury severity, timing thresholds, and outcome definitions, a meta-analysis was not feasible. Therefore, a narrative synthesis was conducted, focusing on key endpoints such as mortality, complication rates, ICU and hospital stay, and recovery outcomes. Where possible, subgroup trends were noted, including the influence of timing in hemodynamically stable versus unstable patients and challenges faced in LMICs. This approach provided a clearer understanding of the potential benefits and limitations of early surgical intervention in multisystem trauma.

Results

Study Selection Process

**Figure 1:**
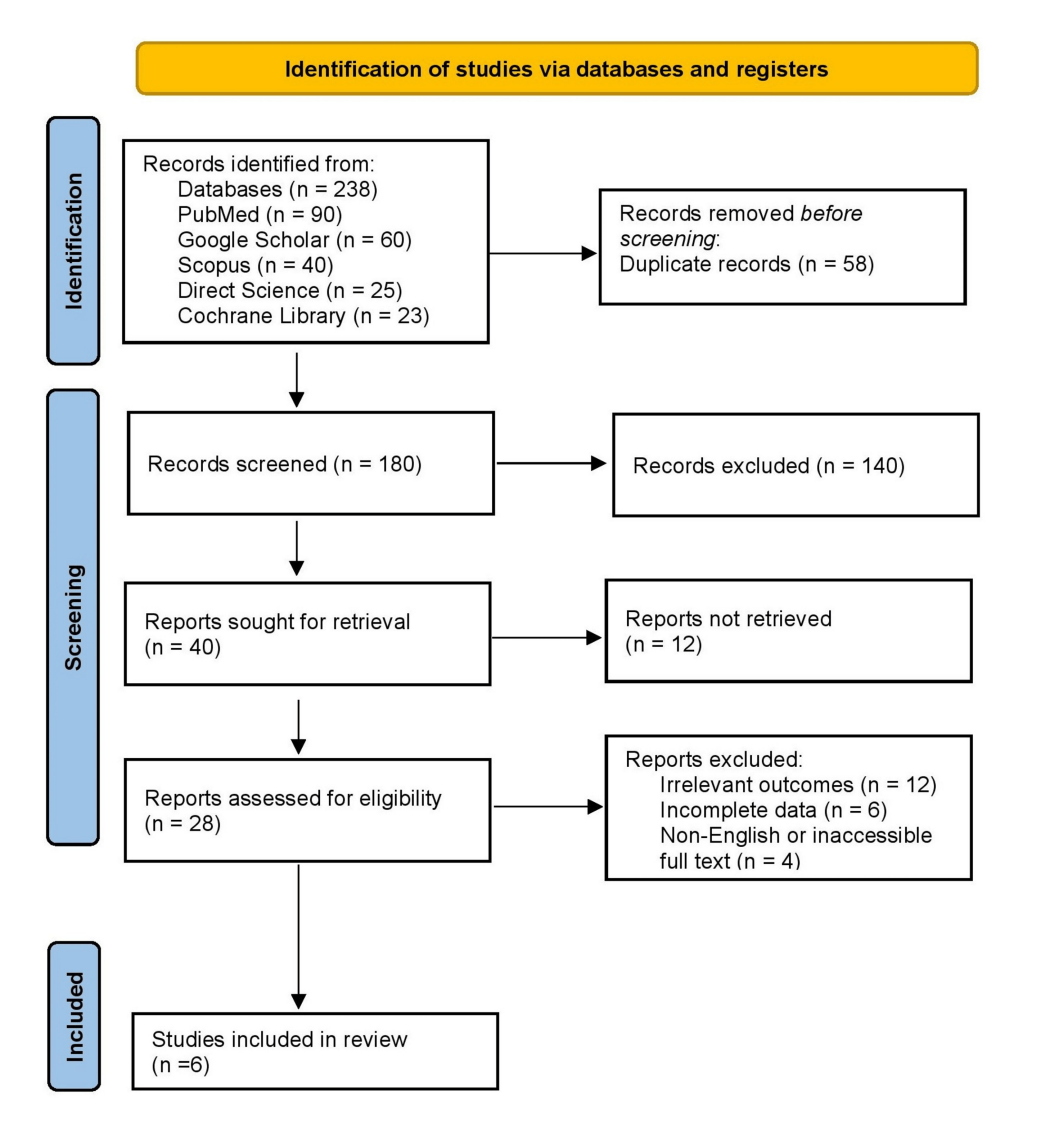
PRISMA flow diagram outlining the study selection process PRISMA: Preferred Reporting Items for Systematic Reviews and Meta-Analyses

Figure 1 presents the PRISMA flow diagram outlining the study selection process. Out of 238 records identified across databases, 58 duplicates were removed, leaving 180 records for title and abstract screening. Of these, 140 were excluded due to irrelevant populations, lack of early versus delayed surgical comparison, or unrelated outcomes. The full texts of 40 articles were then reviewed, with 34 excluded for reasons such as small sample size (<10 patients), non-general surgical procedures, reviews, or non-English publications. In total, six studies met all eligibility criteria and were included in the final review. This rigorous selection process ensured that only relevant, high-quality studies comparing early versus delayed surgical interventions in multisystem trauma patients were synthesized.

Characteristics of the Selected Studies

Table 1 summarizes the key characteristics of the six studies included in this systematic review. The studies encompass a range of designs, including prospective and retrospective cohort studies, a multicenter analysis, a meta-analysis, and a narrative review, reflecting diverse trauma populations and surgical strategies. Sample sizes varied widely, from 91 patients in a single-center cohort to 269,959 cases in the large National Surgical Quality Improvement Program (NSQIP) database analysis. Surgical timing was consistently classified as early (within 24-48 hours of admission) or delayed (beyond 48 hours). The outcomes evaluated across these studies included mortality, ICU and hospital length of stay, postoperative complications such as pneumonia and ARDS, functional recovery, and survival trends based on intervention timing.

**Table 1:** Key characteristics of the studies included in this systematic review TBI – Traumatic Brain Injury; GOS – Glasgow Outcome Score; ACS NSQIP – American College of Surgeons National Surgical Quality Improvement Program; ARDS – Acute Respiratory Distress Syndrome; RCT – Randomized Controlled Trial; ICU – Intensive Care Unit; WEST – Western Trauma Association Criteria

Study (Author, Year)	Study Design	Sample Size	Early Intervention (n)	Delayed Intervention (n)	Outcomes Reported
Wang et al., 2007 [11]	Prospective Cohort (TBI with fractures)	91	46	45	Neuropsychological outcomes, Glasgow Outcome Score, pneumonia incidence, hospital stay
Franklin et al., 2024 [12]	Retrospective Cohort (NSQIP analysis)	269,959	192,550	77,409	30-day mortality, serious morbidity, all morbidity
Dormann et al., 2025 [13]	Observational Cohort	418	210	208	ARDS, pneumonia incidence
Steinfeld et al., 2024 [14]	Meta-analysis of RCTs & Cohorts	335 patients	Not applicable	Not applicable	ICU admission, ICU length of stay, respiratory complications, mortality
Pape et al., 2009 [15]	Narrative Review of Damage Control vs Early Total Care	Not applicable	Not applicable	Not applicable	Classification of stable vs unstable patients, timing recommendations, morbidity/mortality trends
Tsai et al., 2024 [16]	Retrospective Multicenter Cohort	1,015	520	495	In-hospital mortality, ICU stay, survival by WEST time

Risk of Bias Assessment

As shown in Table 2, the narrative review by Pape et al. was rated low risk using SANRA [15], despite inherent limitations of non-systematic reviews. The cohort studies (Wang et al. [11], Franklin et al. [12], Dormann et al. [13], and Tsai et al. [16]), assessed with ROBINS-I, were judged to have moderate risk due to potential confounding and protocol variability. The meta-analysis by Steinfeld et al. [14], evaluated with AMSTAR 2, was also rated moderate risk owing to possible heterogeneity and publication bias, though its methodology was reliable.

**Table 2:** Risk of bias assessment of the included studies ROBINS-I – Risk Of Bias In Non-randomized Studies – of Interventions; AMSTAR 2 – A MeaSurement Tool to Assess Systematic Reviews 2; SANRA – Scale for the Assessment of Narrative Review Articles; RCT – Randomized Controlled Trial

Study (Author, Year)	Study Design	Tool Used	Risk of Bias Assessment	Key Concerns
Wang et al., 2007 [11]	Prospective Cohort	ROBINS-I (Risk Of Bias In Non-randomized Studies – of Interventions)	Moderate	Potential confounding, lack of blinding
Franklin et al., 2024 [12]	Retrospective Cohort	ROBINS-I	Moderate	Selection bias, unmeasured confounders
Dormann et al., 2025 [13]	Observational Cohort	ROBINS-I	Moderate	Protocol variability, retrospective data limits
Steinfeld et al., 2024 [14]	Meta-analysis of RCTs & Cohorts	AMSTAR 2 (A MeaSurement Tool to Assess Systematic Reviews 2)	Moderate	Heterogeneity, potential publication bias
Pape et al., 2009 [15]	Narrative Review	SANRA (Scale for the Assessment of Narrative Review Articles)	Low	Non-systematic nature of review
Tsai et al., 2024 [16]	Retrospective Multicenter Cohort	ROBINS-I	Moderate	Data heterogeneity, potential residual confounding

Discussion

This systematic review emphasizes the consistent advantages of early definitive surgical intervention in multisystem trauma patients. Large-scale evidence, particularly from the ACS NSQIP cohort and the multicenter study by Franklin et al. [12], indicates that early surgery (within 24-48 hours of admission) is linked to lower mortality rates, fewer postoperative complications, and reduced ICU and hospital length of stay. These outcomes are likely attributable to the rapid control of hemorrhage and contamination, as well as the prevention of systemic inflammatory escalation, which can lead to sepsis or multi-organ failure [17]. These findings strongly support early surgical management, especially in hemodynamically stable patients who can tolerate definitive interventions. However, not all patients benefit from an early surgical approach, as highlighted by Pape et al.’s [15] narrative review, which advocates for the damage control orthopedics paradigm in unstable polytrauma cases. Wang et al. [11] and Dormann et al. [13] also noted that, in patients with severe physiological derangements, a delayed approach following initial stabilization can be beneficial, allowing time for correction of acidosis, coagulopathy, and hypothermia. Therefore, while early surgery remains the ideal in most cases, individualized assessment based on trauma severity and physiological stability remains paramount [18].

Another critical factor influencing outcomes is the heterogeneity of study designs and definitions of “early” versus “delayed” surgery. The meta-analysis by Steinfeld et al. [12] highlights the variability in timing thresholds, patient selection, and outcome reporting, which complicates the ability to draw universally applicable conclusions. Additionally, functional recovery outcomes, such as neuropsychological performance and quality of life, were not uniformly assessed across studies, leaving gaps in understanding the long-term benefits of early intervention. These inconsistencies call for the adoption of standardized protocols and clearly defined endpoints in future trauma research.

Finally, global disparities in trauma care significantly affect the feasibility of early surgical strategies. In LMICs, delays in surgical management often result from systemic barriers, including inadequate infrastructure, limited workforce, and resource constraints, rather than clinical decision-making. These challenges underline the need for capacity-building and resource allocation to make early surgical care accessible across diverse healthcare settings [19]. Moving forward, multicenter collaborative trials and international consensus guidelines will be critical in refining surgical timing strategies for multisystem trauma patients.

## Conclusions

Early definitive surgery, including general surgery such as laparotomy, bowel repair, or splenectomy within 24-48 hours, is linked to reduced mortality, postoperative complications, and hospital stays in multisystem trauma patients. However, surgical timing must remain flexible as per protocol for hemodynamically unstable or coagulopathic patients often benefit from an initial damage‑control laparotomy, followed by definitive repair once stabilized. The wide variation in operative protocols and resource availability across institutions highlights the need for consensus-driven guidelines and multicenter trials to establish clear, general surgery-specific timing recommendations.
